# Aspects of Mathematical Modelling of Pressure Retarded Osmosis

**DOI:** 10.3390/membranes6010013

**Published:** 2016-02-03

**Authors:** Yuri G. Anissimov

**Affiliations:** School of Natural Sciences, Griffith University, Parklands Drive, Southport 4222, Australia; y.anissimov@griffith.edu.au; Tel.: +61-7-555-28496; Fax: +61-7-555-28065

**Keywords:** pressure retarded osmosis, mathematical modelling, energy generation

## Abstract

In power generating terms, a pressure retarded osmosis (PRO) energy generating plant, on a river entering a sea or ocean, is equivalent to a hydroelectric dam with a height of about 60 meters. Therefore, PRO can add significantly to existing renewable power generation capacity if economical constrains of the method are resolved. PRO energy generation relies on a semipermeable membrane that is permeable to water and impermeable to salt. Mathematical modelling plays an important part in understanding flows of water and salt near and across semipermeable membranes and helps to optimize PRO energy generation. Therefore, the modelling can help realizing PRO energy generation potential. In this work, a few aspects of mathematical modelling of the PRO process are reviewed and discussed.

## 1. Introduction

When fresh river water mixes with salty sea water, energy equal to the one that is produced in a waterfall about 200 meters high is lost [1]. If only 25%–30% of this energy is converted to electricity it will be equivalent to damming a river with a hydroelectric dam with a height of about 60 meters. The concept of using salinity gradient for power generation was first introduced by Pattle [1] in 1954 who described “hydroelectric pile” apparatus (now called reversed electrodialysis) to harness this energy. Hitherto, it appears that a method based on osmosis, proposed by Loeb in 1973 and first published in 1975 [2], is closer to practical realization. The first, and hitherto the only power plant prototype based on pressure retarded osmosis (PRO) was commissioned by Norwegian state-owned power company Statkraft in 2009 and is now in operation.

**Figure 1:**
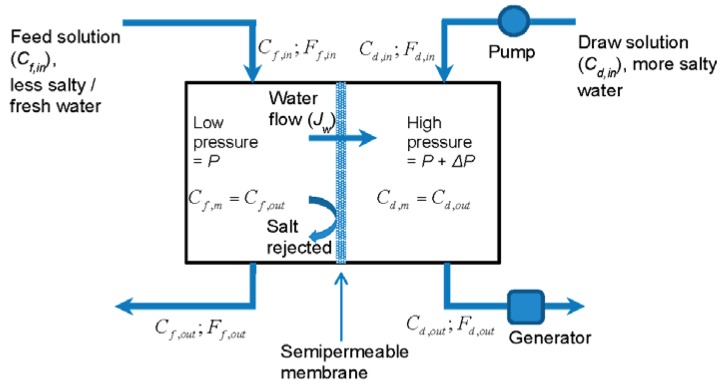
Schematic representation of a classic pressure retarded osmosis (PRO) energy generation plant with a compartmental geometry (adapted from [3]). The feed and draw compartments are assumed to be well stirred, so that the concentration at the membrane surface is the same as the outflowing concentration: $C_{f,out}=C_{f,m}$ and $C_{d,out}=C_{d,m}$.

Generating energy based on PRO relies on creating a pressure in a more salty draw solution using the osmotic flow of water from a less salty feed solution through a semipermeable membrane (see Figure 1). Energy is produced by a generator using the “excess” pressure and flow due to the osmotic flow of water.

PRO energy generation has recently been extended to include closed loop systems, for example, one based on ammonia-carbon dioxide [4] and novel PRO coupled with electric power generation from the electrokinetic streaming potential [5]. A PRO system capable of utilizing low-grade heat not recoverable with existing technologies was presented and investigated [6]. For up to date information on these new developments as well as the applicability and practical viability and current limitations of PRO systems readers are referred to recent comprehensive reviews [7,8,9,10,11].

This short review aims to describe mathematical modelling most relevant to energy generation using PRO. Mathematical modelling where analytical approach is possible is given a priority with mathematical derivations somewhat more detailed than usual in the PRO literature. It is hoped that this detailed description of mathematical solutions will be beneficial for students and scientists new to this area of research who need to understand the details of mathematical modelling.

## 2. Ideal Membrane

### 2.1. Compartmental Configuration

The power output of the PRO power generation scheme presented in Figure 1 is the difference between the power produced by the generator (*W_g_*) and that used by the pump (*W_p_*). Assuming that the pressure difference across the pump and the generator (ΔP, also called working pressure) is the same and is equal to the hydrostatic pressure difference across the membrane, the generator and pump powers can be expressed as: $W_{g}=\left(S_{m}J_{w}+F_{d,in}\right)\Delta P$ and $W_{p}=F_{d,in}\Delta P$ respectively, where $J_{w}$ is the osmotic water flux, $S_{m}$ is the surface area of the membrane and $F_{d,in}$ is the draw solution pump flow rate or flow rate into the draw compartment. Therefore, the power generated per unit area of the membrane ($W=\left(W_{g}-W_{p}\right)/S_{m}$) is [3]:

(1) $$W=J_{w}\Delta P$$

The osmotic water flux is positive (that is, from the feed to the draw solution) if the osmotic pressure difference across the membrane (Δπ) is greater than the hydrostatic pressure (Δπ>ΔP) and is linearly proportional to the difference between the osmotic and hydrostatic pressures [3]:

(2) $$J_{w}=A_{w}(\Delta \pi -\Delta P)$$

where $A_{w}$ is the water permeation coefficient of the semipermeable membrane. It is important to note that Equation (1) does not account for losses due to the low pressure water pumping (feed solution side) required for the operation of a PRO plant and inefficiencies of converting energy at the generator and the high pressure pump (draw solution side).

Combining Equations (1) and (2) yields:

(3) $$W=A_{w}\left(\Delta \pi -\Delta P\right)\Delta P$$

Since the hydrostatic pressure (ΔP) can be varied, *W* can be maximized with regard to ΔP. Differentiating Equation (3) with respect to ΔP and finding where the derivative of power is zero ($W\prime =A_{w}\left(\Delta \pi -2\Delta P\right)=0$) gives optimal hydrostatic pressure: ΔP=Δπ/2. Therefore, the maximum power is:

(4) $$W_{\text{max}}=A_{w}\frac{\Delta \pi^{2}}{4}$$

Equation (4) indicates ways to increase power output of PRO, which is possible by selecting membranes with higher permeability ($A_{w}$), or increasing osmotic pressure difference (Δπ), for example, by increasing salt concentration of the draw solution. Increasing the osmotic pressure difference is especially attractive as dependence of the maximum power in (4) on Δπ is quadratic, but this option can be limited by what solutions are available.

The osmotic pressure difference is determined by the concentration difference across the membrane:

(5) $$\Delta \pi =\pi (C_{d,m})-\pi (C_{f,m})$$

where $C_{f,m}$ and $C_{d,m}$are concentrations of salt on the feed and draw sides of the membrane respectively and π(C) is a monotonically increasing function (see Equation (7)). For the compartmental configuration it is assumed that, due to the effective stirring, concentrations near membrane surfaces are the same as bulk concentrations, which in turn are equal to the outflowing concentrations: $C_{f,out}=C_{f,m}$ and $C_{d,out}=C_{d,m}$. Due to the osmotic water flow through the membrane ($J_{w}$), these concentrations are not equal to the feed and draw concentrations flawing into the compartments ($C_{f,in}$) and ($C_{d,in}$) respectively, but can be related to them by considering mass conservation of water ($F_{f,out}=F_{f,in}-S_{m}J_{w}$ and $F_{d,out}=F_{d,in}+S_{m}J_{w}$) and salt ($C_{f,out}F_{f,out}=C_{f,in}F_{f,in}$ and $C_{d,out}F_{d,out}=C_{d,in}F_{d,in}$) in the system, yielding:

(6) $$C_{f,m}=C_{f,out}=C_{f,in}\frac{F_{f,in}}{F_{f,in}-J_{w}S_{m}}\;\text{and}\;C_{d,m}=C_{d,out}=C_{d,in}\frac{F_{d,in}}{F_{d,in}+J_{w}S_{m}}$$

where $F_{f,in}$ and $F_{f,out}$ are the feed solution flow rates into and out of the feed compartment and it is assumed that the draw and feed compartments are well stirred and there is no concentration polarization (see Section 3) at the membrane surface. Increasing the difference between concentrations of the feed and draw sides of the membrane ($C_{d,m}-C_{f,m}$) increases Δπ and therefore $W_{\text{max}}$. The power can be increased further by increasing the feed and draw pump flow rates, as according to Equation (6) this will increase $C_{d,m}-C_{f,m}$. The latter could be counterproductive if losses caused by the increased pumping rates are higher than gains due to the rise in the osmotic pressure.

The osmotic pressure is linearly proportional to a salt concentration (*C*, expressed in moles per volume) when the concentration is not large [12]:

(7) π(C)=iCRT

where *R* is the ideal gas constant, *T* is the absolute temperature and *i* is the dimensionless van't Hoff factor. The van’t Hoff factor for electrolytes depends on the degree of the dissociation and the number of ions. For NaCl solutions of up to the concertation of sea water *i* ≈1.9 was suggested [12].

If the feed and draw concentrations are known, Equations (4) to (7) can be used to calculate the maximum power per unit area of a membrane for a given membrane permeability.

### 2.2. Counterflow Configuration

**Figure 2:**
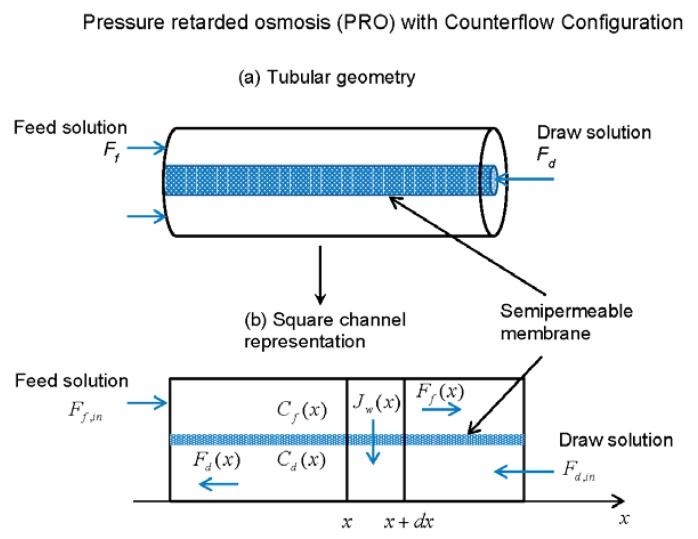
(**a**) A diagram representing tubular geometry and counterflow configuration for PRO. (**b**) Simplified representation of the tubular geometry as a square channel. Concentrations ($C_{d}(x)$ and $C_{f}(x)$) and flows ($J_{w}(x)$, $F_{d}(x)$ and $F_{f}(x)$) are the functions of the position *x* along the channel for the counterflow configuration.

Compartmental geometry is easy to realize and analyze in laboratory conditions and where only a small area of the membrane is necessary. In this case, draw and feed compartments volumes to the surface of the membrane ratio are not small and it is easy to arrange the effective stirring so that the compartments are well stirred. When stirring is effective, the concertation inside the compartments is uniform and practically the same near the input and the output of the draw and feed solutions (top and bottom in Figure 1 respectively). In practical applications of PRO very large areas of membrane are required: millions of square meters of the membrane to generate just tens of megawatts of power. In this case, the effective stirring is not practical and a concentration gradient will form between the input and the output of the draw and feed solutions due to the water flow through the membrane. Schematic diagram of a PRO unit with the counterflow configuration is presented in Figure 2. The tubular membrane shape is perhaps the most practical similar to desalination units where membranes are organized as multiple small bore tubes that are placed in a much larger metal tube casing. The small diameter of the tubes leads to a large surface area of the membrane in a relatively small volume.

In Figure 2b the tubular geometry is replaced with the square channel representation for simplicity and better visualization of the problem. To derive transport equations for this counterflow configuration we will generally follow the approach presented by Sharqawy *et al.* [13], changing to notations consistent with this work. As concentrations and flows in the feed and draw channels are now functions of *x*, where *x* is the distance along the channel, Equation (2) for the water flow across the membrane becomes:

(8) $$J_{w}(x)=A_{w}\left[\pi \left(C_{d}(x)\right)-\pi \left(C_{f}(x)\right)-\Delta P\right]$$

where $C_{d}(x)$, $C_{f}(x)$ and $J_{w}(x)$ are the draw and feed concentrations and the water flow per unit area of the membrane at position *x* along the channel respectively. Here we assumed that the hydrostatic pressure, ΔP, is constant along the channel, even though pressure gradient may form in a thin tube due to the water viscosity, this pressure is likely to be small compared to the hydrostatic pressure. We have also assumed in Equation (8) that concentrations in the feed and draw channels are uniform across the channel. This is only possible if there is no concentration polarization (see Section 3).

We further assume, as for the compartmental geometry, that only the pure water flows through the membrane. Taking into account the pure water flow through the section of the membrane from *x* to x+dx away from the feed to the draw solution the feed flow rate can be expressed as:

(9) $$F_{f}(x)=F_{f}(x+dx)+J_{w}(x)dS_{m}$$

where $dS_{m}$ is the area of the membrane between *x* and *x* + *dx*. This area can be further expressed as $dS_{m}=wdx$, where *w* is the width of the channel or the circumference of the tubular membrane.

Substituting $F_{f}(x+dx)=F_{f}(x)+F_{f}^{\prime }(x)dx$ in Equation (9) yields:

(10) $$\frac{dF_{f}}{dx}=-J_{w}(x)w$$

Using the initial condition $F_{f}(0)=F_{f,in}$ and integrating Equation (10) gives:

(11) $$F_{f}(x)=F_{f,in}-\int_{0}^{x}J_{w}(x)wdx=F_{f,in}-F_{w}(x)$$

where the integral in Equation (11) was replaced with $F_{w}(x)$, the flow of pure water through the membrane from feed to draw solution between 0 and *x*.

A similar consideration for the draw flow rate leads to the differential equation identical to Equation (10), with a change of f→d. Using the initial condition $F_{d}(L)=F_{d,in}$, where *L* is the length of the channel, yields:

(12) $$F_{d}(x)=F_{d,in}+F_{w}(L)-F_{w}(x)$$

Here we assumed for simplicity that $F_{d}(x)$ is positive, even though $F_{d}$ is directed against the positive direction of the *x* axis (Figure 2b).

As there is no salt flow through the membrane the flux of salt for the feed and draw sides is constant: $F_{f}(x)C_{f}(x)=F_{f}(0)C_{f}(0)=F_{f,in}C_{f,in}$ and $F_{d}(x)C_{d}(x)=F_{d}(L)C_{d}(L)=F_{d,in}C_{d,in}$. Using these equations and Equations (11) and (12), concentrations in the feed and draw solutions can be expressed as:

(13) $$C_{f}(x)=\frac{F_{f,in}C_{f,in}}{F_{f,in}-F_{w}(x)};C_{d}(x)=\frac{F_{d,in}C_{d,in}}{F_{d,in}+F_{w}(L)-F_{w}(x)};$$

In Equations (13) parameters $F_{f,in},F_{d,in},C_{f,in}$ and $C_{d,in}$ are operational parameters of the PRO system that are determined, and the only undetermined function is $F_{w}(x)=\int_{0}^{x}J_{w}(x)wdx$. The differential equation for this function can be derived by noting that $F_{w}^{\prime }(x)=J_{w}(x)w$ and using Equations (8) and (13):

(14) $$\frac{dF_{w}}{dx}=A_{w}w\left[\pi \left(\frac{F_{d,in}C_{d,in}}{F_{d,in}+F_{w}(L)-F_{w}(x)}\right)-\pi \left(\frac{F_{f,in}C_{f,in}}{F_{f,in}-F_{w}(x)}\right)-\Delta P\right]$$

Equation (14) is a separable first order differential equation which can be solved by integration:

(15) $$\int_{0}^{F_{w}(x)}\frac{dF_{w}}{\left[\pi \left(\frac{F_{d,in}C_{d,in}}{F_{d,in}+F_{w}(L)-F_{w}}\right)-\pi \left(\frac{F_{f,in}C_{f,in}}{F_{f,in}-F_{w}}\right)-\Delta P\right]}=A_{w}wx$$

The integral in this equation can only be integrated analytically when the osmotic pressure is linearly proportional to a salt concentration (see Equation (7)). In this case the integrand in Equation (15) can be presented as two simple fractions of the form $a/\left(b-F_{w}\right)$, where *a* and *b* are constants, and the integration yields logarithmic expressions (for details see Equation [13]). Even in this simpler linear case it is not possible to represent $F_{w}(x)$ explicitly and numerical approach is required. Sharqawy *et al.* [13] analyzed in detail the linear osmotic pressure case by dimensionalising Equation (15) and introducing notations analogous to a mathematical modelling of heat exchangers. The non-linear osmotic pressure case was also considered for both the counterflow and parallel flow ($F_{f}$ and $F_{d}$ in the same direction) configurations, but it was found that for the case of the seawater as the draw solution and the river water as the feed solution the error of using the linear approximation is not significant [13]. Sharqawy *et al.* [13] also modelled parallel-flow configuration for PRO, but concluded that, just as for the heat exchanges, that the counterflow configuration is more efficient for the same area of the membrane used. Approach similar to work of Sharqawy *et al.* [13] was used to analyze limits of power generation due to finite membrane area [14].

After numerically solving Equation (15), the total power generated by the PRO system ($W_{total}$) can be determined as:

(16) $$W_{total}=F_{w}(L)\Delta P$$

and further analyzed to maximize the power relative to the operational parameters of the PRO system, including the hydrostatic pressure (ΔP). Given the number of parameters, this is not a straightforward exercise and requires a numerical approach.

## 3. Concentration Polarization

**Figure 3:**
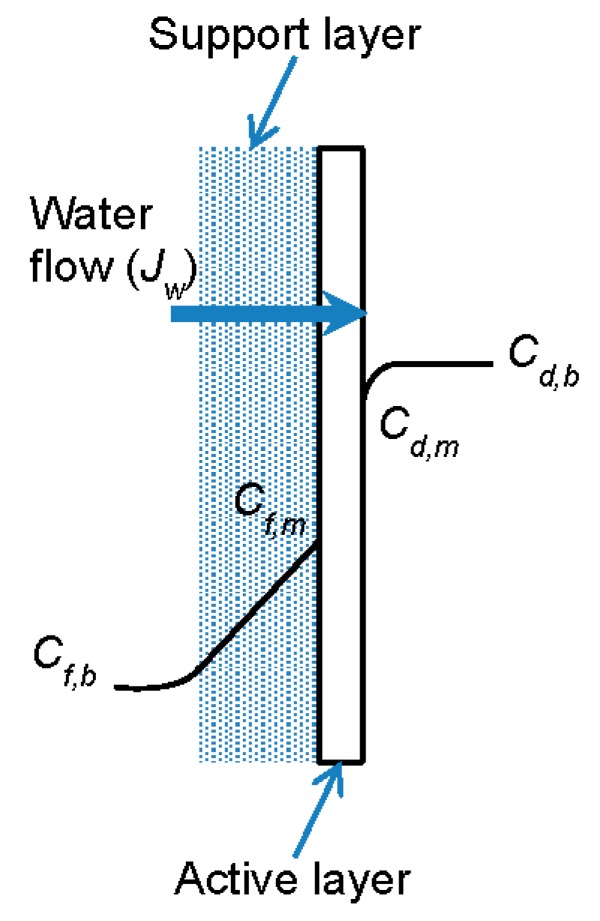
A diagram representing the concentration polarization for an asymmetric membrane with the support layer due to the water flow through the membrane. Here *C_f,m_* and *C_d,m_* are concentrations of salt on the feed and draw sides of the active layer respectively and *C_f,b_* and *C_d,b_* are the bulk concentrations on the feed and draw sides of the membrane respectively.

Concentration polarization is arguably the most significant problem that dramatically reduces the power output of the PRO process and reduces the applicability of equations presented above for the ideal membrane. The polarization had been experimentally investigated and mathematically modelled for the first time more than 30 years ago [3,15]. Experimental work on the polarization was later conducted with more modern membranes [16]. In this review the mathematical modelling of the polarization will be presented for a realistic asymmetric membrane with the porous support layer against the feed solution as shown in Figure 3. The diagram of the concentration polarization is shown in Figure 3 and is represented by the significant increase in the feed solution concentration at the surface of the active layer. This increase is due to the convective transport of salt by the water flow in the support layer of membrane, as the salt cannot penetrate through the active layer of membrane and needs to diffuse against the flow. It is also shown that concentration changes in the unstirred boundary layers near the membrane surfaces (Figure 3). Mathematical modelling of concentration polarization that takes into account the unstirred boundary layers was presented in [17]. The concentration polarization in this case is inversely proportional to the Sherwood number, which in turn depends on the Reynolds number, Schmidt number and geometric dimensions of the channel [18]. Salt leakage/flow across the active layer of the membrane in the direction opposite to the water flow due to the membrane imperfections further increases the concentration polarization.

For simplicity, let’s assume that the concentration changes in the unstirred boundary layers near the membrane surfaces are negligible. Then the boundary condition at the support layer will be: [3]

(17) $$C(x=0)=C_{f,b}$$

where it was assumed that *x* = 0 at the left boundary of the support layer and the positive *x* is in the direction of the osmotic water flow.

The salt flux in the support layer is the sum of diffusive and convective fluxes: [17]

(18) $$J_{s}=-D\varepsilon \frac{dC(x)}{dx}+J_{w}C(x)$$

where *D* is the diffusion coefficient of salt and ε is the porosity of the support layer. For the steady state $J_{s}$ is constant, and Equation (18) is the first order inhomogeneous differential equation with constant coefficients and can be easily integrated yielding:

(19) $$C(x)=\frac{J_{s}}{J_{w}}+E\text{ exp}\left(\frac{J_{w}}{D\varepsilon }x\right)$$

where *E* is the integrating constant which can be determined using boundary condition (Equation (17)), so that:

(20) $$C(x)=\frac{J_{s}}{J_{w}}+\left(C_{f,b}-\frac{J_{s}}{J_{w}}\right)\text{exp}\left(\frac{J_{w}}{D\varepsilon }x\right)$$

The concentration at the feed side of the active layer can now be determined as $C_{f,m}=C(x=\tau t)$, where *t* is the thickness and *τ* the tortuosity of the support layer, therefore:

(21) $$C_{f,m}=\frac{J_{s}}{J_{w}}+\left(C_{f,b}-\frac{J_{s}}{J_{w}}\right)\text{exp}\left(J_{w}K\right)$$

where K=τt/(Dε) is a measure of diffusional resistance to salt transport in the support layer. The support layer structural parameter *S* (=τt/ε) depends only on the microstructure of the support membrane and is now commonly used in the PRO literature [17,19,20] to describe the concentration polarization. It follows from definition of these parameter that *K* = *S*/*D*.

It is appropriate to assume that the salt leakage across the active layer ($J_{s}$) is linearly proportional to the salt concentration difference across the layer: [3]

(22) $$J_{s}=B\left(C_{f,m}-C_{d,m}\right)\approx B\left(C_{f,m}-C_{d,b}\right)$$

where *B* is the salt permeation coefficient across the active layer. Substituting $J_{s}$ from Equations (22) to (21) yields linear equation for $C_{f,m}$ that can be easily solved:

(23) $$C_{f,m}=\frac{C_{f,b}J_{w}\text{exp}\left(J_{w}K\right)+C_{d,b}B\left(\text{exp}\left(J_{w}K\right)-1\right)}{J_{w}+B\left(\text{exp}\left(J_{w}K\right)-1\right)}$$

In this equation, the concentration at the feed side of the active layer is expressed using parameters of the membrane, the bulk salt concentrations and osmotic water flow ($J_{w}$). Equation (23) together with Equations (2) and (5) form an explicit set of equations which allow determination of $J_{w}$ from parameters of the membrane, the bulk salt concentrations and the hydrostatic pressure (ΔP). These equations generally need to be solved numerically.

Lee *et al.* [3] using equations similar to Equation (23), Equation (2) and Equation (5), assuming the linear relationship between concentration and osmotic pressure (Equation (7)) with pure water as a feed solution ($C_{f,b}=0$) and zero hydrostatic pressure (ΔP=0) derived expression for the diffusional resistance (*K*):

(24) $$K=\frac{1}{J_{w0}}\text{ln}\left(\frac{A_{w}\pi \left(C_{d,b}\right)-J_{w0}}{B}+1\right)$$

where $J_{w0}$ is experimentally measured water flow for $C_{f,b}=0$ and ΔP=0.

The mathematical approach is very similar when the concentration changes in the unstirred boundary layers near the membrane surfaces are not negligible. Readers are referred to works by Yip *et al.* [17] and McCutcheon *et al.* [18,21] for equations in this case.

## 4. Module-Scale Analysis of PRO

In Section 2.1 the optimal hydrostatic pressure for an ideal membrane with simple compartmental geometry was determined. For realistic osmotic membranes with significant concentration polarization the process of finding the optimal hydrostatic pressure in general involves a numerical solution with iterations [22,23]. The problem becomes even more challenging for module-scale analysis of PRO when concentration polarization has to be combined with the realistic flow and membrane configurations. Numerical modelling of parallel-flow configuration for PRO was given for the case of concentration polarization [24]. In this work authors introduced a two-dimensional model similar to that presented for crossflow microfiltration and ultrafiltration [25].

Module-scale analysis of PRO for counterflow (or counter-current) and parallel-flow (co-current) configurations were recently investigated taking into account the unfavorable effects of reverse salt flux, internal concentration polarization, and external concentration polarization [20]. In this case, the water and salt fluxes can be represented as: [17,20]

(25) $$J_{w}=A_{w}\left\{\frac{\pi_{d}\;\text{exp}\left(-\frac{J_{w}}{k}\right)-\pi_{f}\;\text{exp}\left(\frac{J_{w}S}{D}\right)}{1+\frac{B}{J_{w}}\left[\text{exp}\left(\frac{J_{w}S}{D}\right)-\text{exp}\left(-\frac{J_{w}}{k}\right)\right]}-\Delta P\right\}$$

(26) $$J_{s}=A_{w}\left\{\frac{C_{d}\;\text{exp}\left(-\frac{J_{w}}{k}\right)-C_{f}\;\text{exp}\left(\frac{J_{w}S}{D}\right)}{1+\frac{B}{J_{w}}\left[\text{exp}\left(\frac{J_{w}S}{D}\right)-\text{exp}\left(-\frac{J_{w}}{k}\right)\right]}-\Delta P\right\}$$

where *k* (=*D*/*δ*, *δ* – effective unstirred layer thickness) is the mass transfer coefficient of the draw solution and $\pi_{d},\pi_{f}$ are osmotic pressures corresponding to draw and feed solutions respectively. Yip *et al.* [17] presented detailed derivations of Equations (25) and (26). They also demonstrated that membrane that balances permeability and selectivity allows to achieve the highest potential peak power density for given feed and draw solution concentrations [17].

These equations have to be solved together with the mass transfer equations, which in the case of the counter-current flow operation are: [20]

(27) $$\frac{dF_{d}\left(s\right)}{ds}=J_{w}\left(C_{d}(s),C_{f}(s),\Delta P\right)$$

(28) $$\frac{dF_{f}\left(s\right)}{ds}=J_{w}\left(C_{d}(s),C_{f}(s),\Delta P\right)$$

(29) $$\frac{d\left(F_{d}\left(s\right)C_{d}(s)\right)}{ds}=-J_{s}\left(C_{d}(s),C_{f}(s),\Delta P\right)$$

(30) $$\frac{d\left(F_{f}\left(s\right)C_{f}(s)\right)}{ds}=-J_{s}\left(C_{d}(s),C_{f}(s),\Delta P\right)$$

where *s* is the relative position along the module represented as membrane area from the draw solution entrance to the position in the module and normalized to the total area of the membrane (*S_m_*). Note that Equation (28) is similar to Equation (10), but has the positive sign in front of *J_w_*, as the direction of *x* selected for Equation (10) is opposite to that for *s* in Equation (28). The boundary conditions for Equations (27)–(30) are $F_{d}\left(0\right)=F_{d,in}$, $F_{f}\left(1\right)=F_{f,in}$, $C_{d}\left(0\right)=C_{d,in}$ and $C_{f}\left(1\right)=C_{f,in}$ [20].

Straub *et al.* [20] numerically solved Equations (25)–(30) and analyzed power density ($PD=J_{w}\Delta P$, the power generated per membrane area) and the specific energy ($SE=\Delta P\Delta F/\left(F_{f,in}+F_{d,in}\right)$, the energy extracted per total volume of the feed and draw solutions combined) using simplifying assumptions of no pressure loss due to pumping of solutions through the feed and draw channels and no inefficiencies in the pressure exchanger or turbine. The approach allows optimizing operating conditions of a realistic PRO system. It also allowed to determine that the maximum specific energy for the current commercial membranes is 1.1 W/m^2^, only 15% of the power density available for the small scale compartmental (coupon scale) PRO system [20] and well short of 5 W/m^2^ necessary to produce osmotic power on commercial basis [26].

## 5. Other Aspects of Mathematical Modelling of PRO

Another potentially practical configuration for PRO energy system is a spiral wound module [27,28]. The experimental and mathematical modelling research for this configuration was conducted by Xu *et al.* [28]. The dilution of the bulk draw solution for this case is to some extent similar to the counterflow configuration but is further complicated by two different flow paths, axial and spiral [27].

An important aspect of PRO analysis not covered in this review is the thermodynamic efficiency of a PRO process in terms of energy extraction of the Gibbs free energy of mixing. Readers are referred to works by the Elimelech group [29,30] for comprehensive analyses.

Another important factor in realistic module-scale PRO systems are feed and draw channels’ geometry and a pressure loss due to viscose flow of solutions through the channels. Seppälä and Lampinen [31] derived transport equation for osmosis inside a hollow cylindrical fiber, taking into account the cylindrical geometry and hydrostatic pressure drop in the fiber. They solved the equations numerically and found the optimal values of the initial hydrostatic pressure difference between the feed and draw sides of the fiber [31].

In this review, only concentration of a single solute was considered. Such modelling only fully applies for non-dissociating solutes like glucose. In reality PRO systems are most likely to be based on sea and river waters [2] which have multi-ionic composition. In most cases considering NaCl is sufficient to model operation of the PRO systems [24,32], but as NaCl dissociates in water to Na^+^ and Cl^−^ ions, that creates two ionic species which can differ in their permeability through the membrane. In general, solution diffusion and electro-migration have to be taken into account for multi-ionic systems [33]. Yaroshchuk *et al.* [33] considered mathematical modelling of transport of multiple ions where diffusion was coupled to electro-migration and concluded that such modelling was important to understand phenomenon such as negative rejection for some ions in particular that spontaneously arising electric fields may yield much higher NaCl rejection, which could be relevant for energy generating PRO systems.

It is clear from discussions in this work that the mathematical problem of optimizing realistic PRO systems is quite challenging. To help with this optimization Sivertsen *et al.* [12] introduced Iso-watt diagrams that are relatively easy to understand and use, and allow an evaluation of power per unit area of the membrane on basis of membrane characteristics. These diagrams are generated for realistic membranes with concentration polarization and are a useful tool in optimizing PRO energy generating systems, but so far have not been developed to apply to cases of counterflow configurations.

## 6. Conclusions

Mathematical modelling plays an important part in experimental analysis, development and optimization of PRO energy generating systems. In this work the mathematical modelling for compartmental and counterflow configurations were reviewed and presented in some detail for the simple case of an ideal membrane without concentration polarization. The equations presented in this work for these configurations can be used for getting some insight into optimizing energy generation through varying parameters of the system, but are limited to an ideal membrane. Operating realistic PRO systems leads to a very significant concentration polarization, especially in the support layer of a membrane. Basic approach to mathematical modelling of the concentration polarization and main concepts were reviewed. Although there are approaches that allow optimization of parameters of a PRO system with the concentration polarization for the simple compartmental configuration, more modelling work is required to consider the more practical counterflow configuration and concentration polarization.

## References

[1] Pattle R.E. (1954). Production of electric power by mixing fresh and salt water in the hydroelectric pile. Nature.

[2] Loeb S. (1975). Osmotic power-plants. Science.

[3] Lee K.L., Baker R.W., Lonsdale H.K. (1981). Membranes for power-generation by pressure-retarded osmosis. J. Membr. Sci..

[4] McGinnis R.L., McCutcheon J.R., Elimelech M. (2007). A novel ammonia-carbon dioxide osmotic heat engine for power generation. J. Membr. Sci..

[5] Hon K.C., Zhao C.L., Yang C., Low S.C. (2012). A method of producing electrokinetic power through forward osmosis. Appl. Phys. Lett..

[6] Lin S.H., Yip N.Y., Cath T.Y., Osuji C.O., Elimelech M. (2014). Hybrid pressure retarded osmosis-membrane distillation system for power generation from low-grade heat: Thermodynamic analysis and energy efficiency. Environ. Sci. Technol..

[7] Helfer F., Lemckert C., Anissimov Y.G. (2014). Osmotic power with pressure retarded osmosis: Theory, performance and trends—A review. J. Membr. Sci..

[8] Zhao S.F., Zou L., Tang C.Y.Y., Mulcahy D. (2012). Recent developments in forward osmosis: Opportunities and challenges. J. Membr. Sci..

[9] Altaee A., Zaragoza G., Sharif A. (2014). Pressure retarded osmosis for power generation and seawater desalination: Performance analysis. Desalination.

[10] Altaee A., Sharif A. (2015). Pressure retarded osmosis: Advancement in the process applications for power generation and desalination. Desalination.

[11] Straub A.P., Deshmukh A., Elimelech M. (2016). Pressure-retarded osmosis for power generation from salinity gradients: Is it viable?. Energy Environ. Sci..

[12] Sivertsen E., Holt T., Thelin W.R., Brekke G. (2015). Iso-watt diagrams for evaluation of membrane performance in pressure retarded osmosis. J. Membr. Sci..

[13] Sharqawy M.H., Banchik L.D., Lienhard J.H. (2013). Effectiveness-mass transfer units (epsilon-mtu) model of an ideal pressure retarded osmosis membrane mass exchanger. J. Membr. Sci..

[14] Banchik L.D., Sharqawy M.H., Lienhard J.H. (2014). Limits of power production due to finite membrane area in pressure retarded osmosis. J. Membr. Sci..

[15] Mehta G.D., Loeb S. (1978). Internal polarization in the porous substructure of a semipermeable membrane under pressure-retarded osmosis. J. Membr. Sci..

[16] Loeb S., Titelman L., Korngold E., Freiman J. (1997). Effect of porous support fabric on osmosis through a loeb-sourirajan type asymmetric membrane. J. Membr. Sci..

[17] Yip N.Y., Tiraferri A., Phillip W.A., Schiffrnan J.D., Hoover L.A., Kim Y.C., Elimelech M. (2011). Thin-film composite pressure retarded osmosis membranes for sustainable power generation from salinity gradients. Environ. Sci. Technol..

[18] McCutcheon J.R., Elimelech M. (2006). Influence of concentrative and dilutive internal concentration polarization on flux behavior in forward osmosis. J. Membr. Sci..

[19] Chou S.R., Wang R., Shi L., She Q.H., Tang C.Y., Fane A.G. (2012). Thin-film composite hollow fiber membranes for pressure retarded osmosis (pro) process with high power density. J. Membr. Sci..

[20] Straub A.P., Lin S.H., Elimelech M. (2014). Module-scale analysis of pressure retarded osmosis: Performance limitations and implications for full-scale operation. Environ. Sci. Technol..

[21] McCutcheon J.R., McGinnis R.L., Elimelech M. (2006). Desalination by ammonia-carbon dioxide forward osmosis: Influence of draw and feed solution concentrations on process performance. J. Membr. Sci..

[22] Reimund K.K., McCutcheon J.R., Wilson A.D. (2015). Thermodynamic analysis of energy density in pressure retarded osmosis: The impact of solution volumes and costs. J. Membr. Sci..

[23] Yaroshchuk A. (2015). Optimal hydrostatic counter-pressure in pressure-retarded osmosis with composite/asymmetric membranes. J. Membr. Sci..

[24] Thorsen T., Holt T. (2009). The potential for power production from salinity gradients by pressure retarded osmosis. J. Membr. Sci..

[25] Trettin D.R., Doshi M.R. (1980). Limiting flux in ultrafiltration of macromolecular solutions. Chem. Eng. Commun..

[26] Skilhagen S.E. (2010). Osmotic power—A new, renewable energy source. Desalination Water Treat..

[27] Kim Y.C., Kim Y., Oh D., Lee K.H. (2013). Experimental investigation of a spiral-wound pressure-retarded osmosis membrane module for osmotic power generation. Environ. Sci. Technol..

[28] Xu Y., Peng X.Y., Tang C.Y.Y., Fu Q.S.A., Nie S.Z. (2010). Effect of draw solution concentration and operating conditions on forward osmosis and pressure retarded osmosis performance in a spiral wound module. J. Membr. Sci..

[29] Lin S.H., Straub A.P., Elimelech M. (2014). Thermodynamic limits of extractable energy by pressure retarded osmosis. Energy Environ. Sci..

[30] Yip N.Y., Elimelech M. (2012). Thermodynamic and energy efficiency analysis of power generation from natural salinity gradients by pressure retarded osmosis. Environ. Sci. Technol..

[31] Seppala A., Lampinen M.J. (1999). Thermodynamic optimizing of pressure-retarded osmosis power generation systems. J. Membr. Sci..

[32] Achilli A., Cath T.Y., Childress A.E. (2009). Power generation with pressure retarded osmosis: An experimental and theoretical investigation. J. Membr. Sci..

[33] Yaroshchuk A., Bruening M.L., Bernal E.E.L. (2013). Solution-diffusion-electro-migration model and its uses for analysis of nanofiltration, pressure-retarded osmosis and forward osmosis in multi-ionic solutions. J. Membr. Sci..

